# Poly(ethylene glycol)-Alendronate-Coated Magnetite Nanoparticles Do Not Alter Cardiovascular Functions and Red Blood Cells’ Properties in Hypertensive Rats

**DOI:** 10.3390/nano11051238

**Published:** 2021-05-07

**Authors:** Viktoriia Oleksa, Iveta Bernátová, Vitalii Patsula, Silvia Líšková, Peter Bališ, Jana Radošinská, Andrea Mičurová, Michal Kluknavský, Tomáš Jasenovec, Dominika Radošinská, Hana Macková, Daniel Horák

**Affiliations:** 1 Institute of Macromolecular Chemistry, Czech Academy of Sciences, Heyrovského Nám. 2, 162 06 Prague, Czech Republic; oleksa@imc.cas.cz (V.O.); patsula@imc.cas.cz (V.P.); mackova@imc.cas.cz (H.M.); 2 Institute of Normal and Pathological Physiology, Centre of Experimental Medicine, Slovak Academy of Sciences, Sienkiewiczova 1, 813 71 Bratislava, Slovakia; iveta.bernatova@savba.sk (I.B.); silvia.liskova@savba.sk (S.L.); peter.balis@savba.sk (P.B.); andrea.micurova@savba.sk (A.M.); michal.kluknavsky@savba.sk (M.K.); 3 Institute of Pharmacology and Clinical Pharmacology, Faculty of Medicine, Comenius University, Sasinkova 4, 811 08 Bratislava, Slovakia; 4 Institute of Physiology, Faculty of Medicine, Comenius University, Sasinkova 2, 813 72 Bratislava, Slovakia; jana.radosinska@fmed.uniba.sk (J.R.); tomas.jasenovec@fmed.uniba.sk (T.J.); 5 Institute for Heart Research, Centre of Experimental Medicine, Slovak Academy of Sciences, Dúbravská Cesta 9, 841 04 Bratislava, Slovakia; 6 Department of Molecular Biology, Faculty of Natural Sciences, Comenius University, Mlynská Dolina, Ilkovičova 6, 842 15 Bratislava, Slovakia; dominikaradosinska@gmail.com

**Keywords:** magnetic, alendronate, nanoparticles, cardiovascular, red blood cells

## Abstract

In this study, magnetite nanoparticles were prepared and coated with poly(ethylene glycol) terminated by alendronate to ensure firm binding to the iron oxide surface. Magnetic nanoparticles, designated as magnetite coated with poly(ethylene glycol)-alendronate (Fe_3_O_4_@PEG-Ale), were characterized in terms of number-average (*D*_n_) and hydrodynamic (*D*_h_) size, ζ-potential, saturation magnetization, and composition. The effect of particles on blood pressure, vascular functions, nitric oxide (NO), and superoxide production in the tissues of spontaneously hypertensive rats, as well as the effect on red blood cell (RBC) parameters, was investigated after intravenous administration (1 mg Fe_3_O_4_/kg of body weight). Results showed that Fe_3_O_4_@PEG-Ale particles did negatively affect blood pressure, heart rate and RBC deformability, osmotic resistance and NO production. In addition, Fe_3_O_4_@PEG-Ale did not alter functions of the femoral arteries. Fe_3_O_4_@PEG-Ale induced increase in superoxide production in the kidney and spleen, but not in the left heart ventricle, aorta and liver. NO production was reduced only in the kidney. In conclusion, the results suggest that acute intravenous administration of Fe_3_O_4_@PEG-Ale did not produce negative effects on blood pressure regulation, vascular function, and RBCs in hypertensive rats.

## 1. Introduction

Iron oxide-based magnetic nanoparticles (NPs) exhibiting superparamagnetic properties due to their nanoscale size are promising in a variety of bioapplications [1,2]. Such particles were already approved by the Food and Drug Administration (FDA) for magnetic resonance imaging (MRI) of sentinel lymph nodes, liver, spleen, and bowel [3] or treatment of iron deficiency [4]. Examples of commercial polysaccharide-coated magnetic nanoparticles involve Lumirem^®^, Feridex^®^, Endorem^TM^, Feraheme^®^, and GastroMARK^®^ [5]. However, some of them were later withdrawn from the market due to the lack of interest in the medical community, rentability of production, and potential health risks. A major concern relates to their toxic effects on the cells, as well as on the living organism, mainly due to possible interference with iron metabolism. Behavior of iron oxide nanoparticles administered intravenously in the organism depends on their physicochemical properties, such as composition, size, ζ-potential, coating, colloidal stability, concentration, etc. They are excluded mostly by kidneys, if size is <6–15 nm [6]; larger particles are excreted via hepatobiliary clearance and some are internalized in the spleen. Elevated amounts of iron were observed also in the lungs, brain, heart, aorta, and other tissues [7]. A recent study showed superparamagnetic poly(ethylene glycol) (PEG)-coated magnetite NPs did not alter blood pressure and plasma corticosterone levels, but produced tissue-dependent changes in nitric oxide (NO) production in normotensive rats [8]. Importantly, iron oxide NPs altered vascular function in terms of enhanced NO-dependent components of acetylcholine-induced endothelium-dependent relaxation. Circulating nanoparticles may potentially influence red blood cells (RBCs) and damage their membranes. As NPs can pass into RBC cytoplasm, NPs can affect the intracellular environment of RBCs.

The two main forms of iron oxide are magnetite (Fe_3_O_4_) and its oxidized analogue maghemite (γ-Fe_2_O_3_). Their selection depends on the purpose of the application. While some authors prefer higher magnetization of magnetite [9,10], others emphasize oxidation stability of maghemite [11]. NPs were frequently modified to achieve stealth behavior against adaptive immune systems and prolonged circulation in the blood stream, and vice versa, to increase attractivity for some specific cells, e.g., the cancer ones [12,13]. Suitable coating also reduces toxicity of NPs associated mainly with oxidative stress, DNA damage or hemolysis [14,15]. Coatings include low-molecular weight, as well as polymer molecules, and various cell lysates to mask nanoparticles in the blood stream. To anchor polymer coatings on particle surface, various ligands were used, including phosphate [16], (bis)phosphonate [17], sulfo [18], and carboxyl groups [19], or polymer was covalently crosslinked on magnetic nanoparticles [20]. Examples of polymers suitable as particle coatings include dextran, carboxydextran [21], PEG [22], and polyvinylpyrrolidone [23], which are FDA-approved and biocompatible. 

Bisphosphonate-containing molecules belong to drugs for treatment of bone illnesses. Alendronate (Ale) is a bisphosphonate medication used for treatment of osteoporosis in women who have undergone menopause, as well as in men, in whom hypertension is frequent comorbidity, as they form complexes with calcium [24]. In addition, endothelial dysfunction is a hallmark of arterial hypertension and any further damage of the endothelium (inner monolayer of the arteries) would worsen the already existing disease state. That is why any NPs used in medical applications, especially when they are administered intravenously, should not damage the endothelium and/or decrease the release of the endothelium-derived relaxing factors (mainly release of NO) or increase the endothelium-derived contracting factors [25]. 

In this study, Ale was used as a ligand for anchoring PEG on the surface of the iron oxide nanoparticles. The advantage of Ale consists in that it is used in human medicine, and it was used previously as an additive to coating of metals due to its generally good chelating properties [26]. We investigated the influence of magnetite NPs coated with PEG-alendronate (Fe_3_O_4_@PEG-Ale) on certain biological parameters, such as blood pressure, heart rate, vascular function and nitric oxide and superoxide production in the organs and tissues of spontaneously hypertensive rats. In addition, we determined red blood cell fundamental physiological parameters—deformability, osmotic resistance and NO production.

## 2. Materials and Methods

### 2.1. Materials

Sodium salt of (4-amino-1-hydroxy-1-phosphonobutyl)phosphonic acid trihydrate (alendronate; Ale) was purchased from TCI (Tokyo, Japan). α-Methoxy poly(ethylene glycol) succinimidyl ester (PEG-NHS; molar mass  = 5000 g/mol) was purchased from Rapp Polymere (Tuebingen, Germany). Ferric chloride hexahydrate was purchased from Sigma-Aldrich (St. Louis, MO, USA). Ammonium hydroxide and Na_2_HPO_4_·12∙H_2_O and KH_2_PO_4_ used for the preparation of 0.5 M phosphate buffer (PB) were obtained from Lach-Ner (Neratovice, Czech Republic). Ultrapure Q-water that was ultrafiltered using a Milli-Q Gradient A10 system (Millipore, Molsheim, France) was used in all experiments. 

### 2.2. Synthesis of PEG-Alendronate

**Figure 1:**
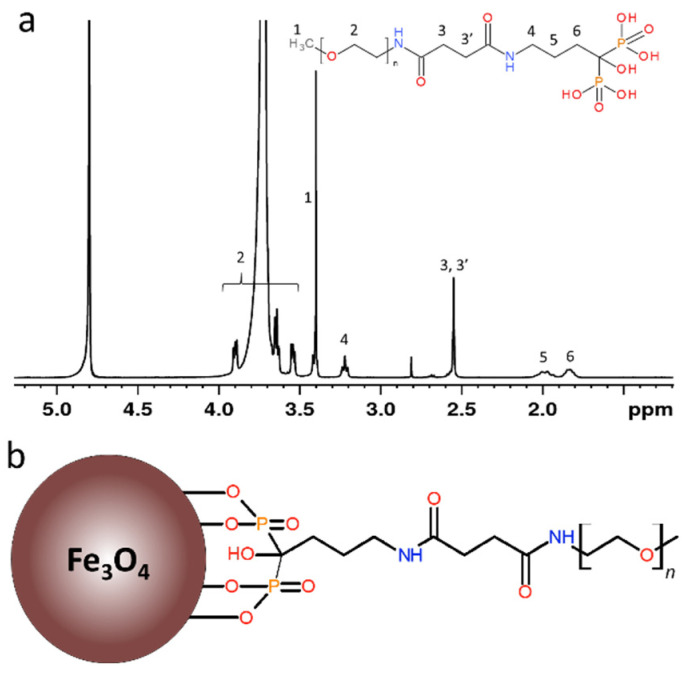
(**a**) ^1^H NMR spectrum of PEG-Ale and (**b**) scheme of Fe_3_O_4_@PEG-Ale nanoparticles. PEG-Ale, poly(ethylene glycol)-alendronate.

Ale (0.14 g) was dissolved in 0.5 M PB (2 mL; pH 7.4) at 0 °C and pH of the solution was adjusted to 7.4 by addition of 4 M aqueous NaOH. PEG-NHS (0.5 g) was then added and the reaction mixture was stirred at 0 °C for 6 h and at room temperature for 16 h. The mixture was acidified with 4 M HCl to pH 2, PEG-alendronate (PEG-Ale) was extracted with CH_2_Cl_2_ (3 × 8 mL) and then combined organic layers were filtered through a 0.45 µm polytetrafluoroethylene filter (Millet, Milwaukee, WI, USA). CH_2_Cl_2_ was removed on a rotary evaporator at 30 °C and the resulting product was vacuum-dried at 60 °C over phosphorus pentoxide. Chemical structure of PEG-Ale in D_2_O was analyzed at 23 °C by ^1^H NMR spectrum (Figure 1a).

### 2.3. Preparation of Fe_3_O_4_ Nanoparticles and Their Modification with PEG-Ale

Aqueous FeCl_3_·6H_2_O (10 mmol) solution (50 mL) was added with vigorous stirring to an iron(II) hydroxide dispersion prepared from aqueous solution (25 mL) of FeCl_2_·4H_2_O (5 mmol) and ammonium hydroxide (40 mmol; 3.7 mL). Resulting black precipitate was magnetically separated and washed with water ten times (100 mL each). Ammonium hydroxide (100 µL) was then added, the product was washed with water three times (50 mL each), and sonicated with a UP400S ultrasonic processor (Hielscher Ultrasonics, Teltow, Germany) for 5 min. To determine magnetic properties and iron oxide concentration, a small part of the colloid was lyophilized. PEG-Ale (22 mg) was then added to an aqueous dispersion of Fe_3_O_4_ nanoparticles (10 mL; 4.4 mg of Fe_3_O_4_/mL) that was then sonicated for 2 min (10% power; Bandeline Sonoplus, Berlin, Germany) and filtered through a sterile 0.45 µm filter to reach a concentration of 4.4 mg of Fe_3_O_4_@PEG-Ale per mL.

### 2.4. Characterization of Nanoparticles

The particles were visualized on a Tecnai Spirit G^2^ transmission electron microscope (TEM; FEI, Brno, Czech Republic). The number-average (*D*_n_), weight-average diameter (*D*_w_), and dispersity (*Ð*) were calculated from TEM micrographs, counting at least 500 individual particles: *D*_n_ = ∑*D*_i_/*N*, *D*_w_ = ∑*D*_i_^4^/∑*D*_i_^3^, *Ð* = *D_w_*/*D*_n,_ where *D*_i_ is the diameter of particle *i* and *N* is the total number of counted particles. Dynamic light scattering (DLS) was measured on a ZEN 3600 Zetasizer Nano Instrument (Malvern Instruments, Malvern, UK) providing hydrodynamic diameter *D*_h_, polydispersity (*PD*), and ζ-potential. Superconducting quantum interference device magnetometry was performed on a Quantum Design MPMS XL device (San Diego, CA, USA). The magnetization curves were measured up to the fields of 3183 kA/m at 5 and 300 K. The zero-field-cooled and field-cooled (ZFC-FC) measurements were carried out in a magnetic field of *H* = 1.59 kA/m. Weight (*M*_w_)- and number-average molar mass (*M*_n_), and polydispersities of the polymers were determined on a Shimadzu high-performance size-exclusion liquid chromatograph (SEC, Tokyo, Japan) equipped with a UV-Vis diode array, OptilabrEX refractive index and DAWN EOS multiangle light scattering detectors (Wyatt, Santa Barbara, CA, USA), and a TSK SuperAW3000 column with methanol/sodium acetate buffer (80/20 *v/v*) eluent (Ph 6.5) at a flow rate of 0.6 mL/min. The ^1^H NMR spectrum was measured by a Bruker Ascend^TM^ 400 spectrometer operating at 400 MHz. Fourier-transform infrared (FTIR) spectrometer (PerkinElmer, Waltham, MA, USA) was equipped with a Specac MKII Golden Gate single attenuated total reflection. Amount of coating was evaluated by a PerkinElmer thermogravimetric analyzer (TGA). The grafting density (*σ*) of PEG on the particle surface was calculated according to Equation (1):

(1) $$\sigma =\frac{(m_{\mathrm{PEG}}/m_{\mathrm{Fe}3O4})\rho \;V{\;N}_{A}}{M_{n}\;S}$$

where *m*_PEG_ and *m*_Fe3O4_ are weight percentages of PEG and magnetite in the particles according to TGA, respectively, *ρ* is magnetite density (5.18 kg/m^3^), *V* is volume of a particle (approximated by the volume of sphere), N*_A_* is Avogadro’s number, *M*_n_ is number-average molar mass of PEG-Ale (5249 g/mol), and *S* is surface area of a particle (approximated by the surface area of sphere). 

### 2.5. Animal Experiments

Male, spontaneously hypertensive rats (SHR) were obtained from the certified animal facility of the Department of Toxicology and Laboratory Animal Breeding, Centre of Experimental Medicine, Dobrá Voda, Slovakia. Rats, 13–16 weeks old, were housed under standard conditions at 22–24 °C and 12-h light/dark cycle and fed with pelleted diet Altromin formula 1320, variant P (Altromin Spezialfutter, Lage, Germany) and tap water ad libitum. All the procedures used in this study were approved by the State Veterinary and Food Administration of the Slovak Republic in accordance with the European Union Directive 2010/63/EU. 

**Figure 2:**
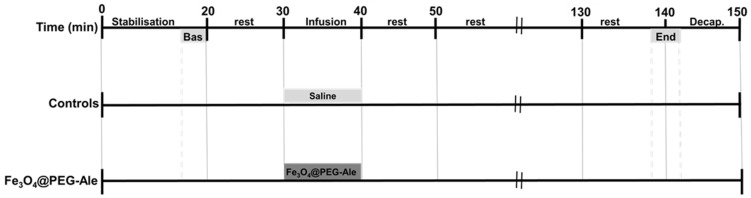
Time course of the experimental protocol. Basal recordings of blood pressure and heart rate were determined at the beginning (Bas) and at the end of the experiment as the average values of ~120-s time periods between 16–20 min and 138–142 min of the experiment. Then, the rats were decapitated (Decap.). Bas, baseline; Fe_3_O_4_@PEG-Ale, Fe_3_O_4_@PEG-alendronate nanoparticles.

Animals were organized into two groups. The control (Cont) group (*n* = 6–7) obtained saline infusion, while the nanoparticle group received Fe_3_O_4_@PEG-Ale nanoparticles (*n* = 5–6). The nanoparticles (1 mg Fe_3_O_4_/kg body weight) dispersed in saline were administered intravenously (IV) for 10 min into the jugular vein. Experimental protocol is shown in the Figure 2. After the experiment, rats were exposed to brief CO_2_ anesthesia and decapitated within 5 min of the final mean arterial pressure (MAP) recording.

### 2.6. Measurement of Blood Pressure and Heart Rate

One day before the experiment, two fine bore polyethylene catheters (Smiths Medical International, Kent, UK) were implanted under 2.5–3% isoflurane anesthesia. One catheter was inserted into the left carotid artery for determination of arterial blood pressure (BP) and heart rate (HR) and the second one was inserted into the jugular vein for IV infusion of NPs or saline as described previously [8]. The experiments were performed in the quiet room to avoid any non-specific stimuli affecting BP and HR, with sampling rate 1 kHz. During the experiments, the conscious rats were placed into a dark plastic box, which allowed their free movement. Arterial catheter was attached to BP recording PowerLab data acquisition system (ADInstruments, Bella Vista, Australia). MAP and HR were recorded during the entire experiment and both parameters were evaluated during 120-s time periods between 10 and 14 min before nanoparticle administration (Bas), during the entire 10 min of NPs infusion, as well as during 120-s time periods ~100 min after the NPs administration (end of the experiment). Results were calculated using LabChart Pro version 8 (ADInstruments, Bella Vista, Australia).

### 2.7. Determination of Activity of Nitric Oxide Synthase

Activity of nitric oxide synthase (NOS) (expressed as pkat/g of protein) was assessed in 20% tissue homogenates by determining [^3^H]-L-citrulline formation from [^3^H]-L-arginine (ARC, St. Louis, MO, USA) as described in detail earlier [8]. Protein concentration was determined using the Lowry method [27].

### 2.8. Production of Superoxide

Tissues (10–25 mg) were placed into ice-cold modified Krebs–Henseleit solution (physiological saline solution, PSS; in mmol/L: 119 NaCl, 4.7 KCl, 1.17 MgSO_4_·7H_2_O, 25 NaHCO_3_, 1.18 KH_2_PO_4_, 0.03 Na_2_EDTA, 2.5 CaCl_2_·2H_2_O, 5.5 glucose). Lucigenin (50 µmol/L), as well as tissue samples alone, were added to PSS bubbled with pneumoxide (5% CO_2_ and 95% O_2_) at 37 °C and pH 7.4 and preincubated in the dark for 20 min. After the preincubation, either background lucigenin-enhanced chemiluminescence or lucigenin-enhanced chemiluminescence produced by tissue samples were measured for 6 min using a TriCarb 2910TR liquid scintillation analyzer (TriCarb, Perkin Elmer, Waltham, MA, USA) [28]. Background counts were subtracted from those of tissue samples and expressed as counts per min per mg of tissue (cpm/mg).

### 2.9. Determination of Vascular Functions

Isolated and cleaned femoral arteries with intact endothelium were cut into segments (two segments of each rat) and placed in Mulvany-Halpern isometric myograph (Dual Wire Myograph system 410A; Danish Myo Technology, Aarhus, Denmark) to investigate vascular function as described in detail previously [8]. Contractions induced by 125 mmol/L K^+^ and serotonin (5-HT; 10^−6^ mol/L) were investigated in the absence and presence of NO synthase inhibitor *N*(ω)-nitro-L-arginine methyl ester (L-NAME, 3 × 10^−4^ mol/L, 30 min pre-incubation). After 20 min of stabilization of 5-HT-induced contraction, endothelium-dependent relaxations were induced by administration of cumulative concentrations of acetylcholine (ACh, 10^−9^ to 10^−5^ mol/L) into the organ chamber. After washing and stabilization of the arteries, NOS inhibitor L-NAME (3 × 10^−4^ mol/L, 30 min incubation time) was added into the organ chamber and ACh-induced relaxations were repeatedly evaluated. Endothelium-independent relaxations produced by vascular smooth muscle cells were investigated using exogenous NO donor sodium nitroprusside (SNP, 10^−9^ to 10^−5^ mol/L). 

### 2.10. Determination of Red Blood Cell Parameters

Red blood cell parameters were determined as described in detail previously [29]. Briefly, RBC deformability assessed by filtration method was expressed as a percentage of RBCs that were able to pass through the filters with pores 5 μm in diameter (Ultrafree-MC SV Centrifugal Filter, Merck Millipore, Ireland). For osmotic resistance, hemolytic assay was applied. RBCs were suspended in solutions with varying concentrations of NaCl (0.1–0.9%), incubated for 30 min and centrifuged. Intensity of hemolysis was determined spectrophotometrically and NaCl concentration in which 50% hemolysis occurred (*IC*_50_) was calculated from obtained data. NO production by RBCs was determined using 4,5-diaminofluorescein diacetate (Abcam, Cambridge, UK). NO dependent fluorescence was observed using a Nikon Eclipse Ti fluorescence microscope (Tokyo, Japan) and quantified using ImageJ software.

### 2.11. Statistical Analyses

Statistical analysis was performed by unpaired or paired Student’s *t*-test, where appropriate. MAP, HR, and vascular functions were analyzed by analysis of variance (ANOVA) for repeated measures. ANOVA analyses were followed by the Bonferroni post hoc test. To assess the difference in RBC parameters before and after the NP administration paired Student’s *t*-test or Wilcoxon test (depending on data normality) were used. The values were found to significantly differ when *p* < 0.05. The data were presented as mean ± standard error of mean (SEM). GraphPad Prism 5.0 (GraphPad Software, San Diego, CA, USA) and Statistica 13.5 (StatSoft, Hamburg, Germany) were used for the statistical analyses.

## 3. Results

### 3.1. Fe_3_O_4_ Nanoparticles Preparation

**Figure 3:**
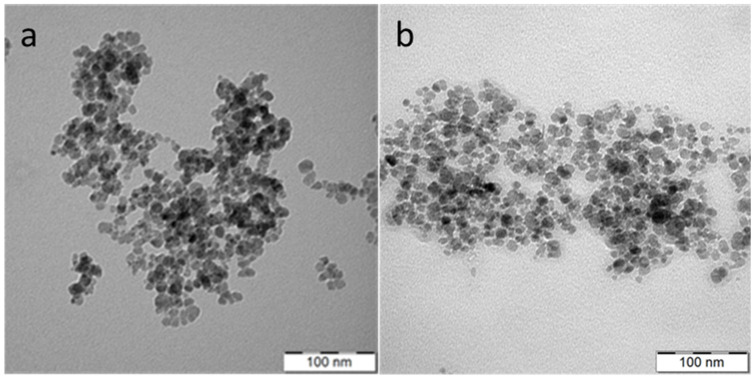
Transmission electron microscope micrographs of (**a**) Fe_3_O_4_ and (**b**) Fe_3_O_4_@PEG-Ale nanoparticles.

Magnetic Fe_3_O_4_ nanoparticles were synthetized by a coprecipitation method with a base. The technique is advantageous due to its simplicity, possibility of large-scale production, and high reaction yield. Resulting Fe_3_O_4_ nanoparticles had number-average diameter *D*_n_ = 11 nm and moderately high dispersity *Ð* = 1.24 according to TEM micrograph (Figure 3a), while hydrodynamic size (*D*_h_ = 100 nm) and polydispersity (*PD* = 0.11) were determined by DLS.

**Figure 4:**
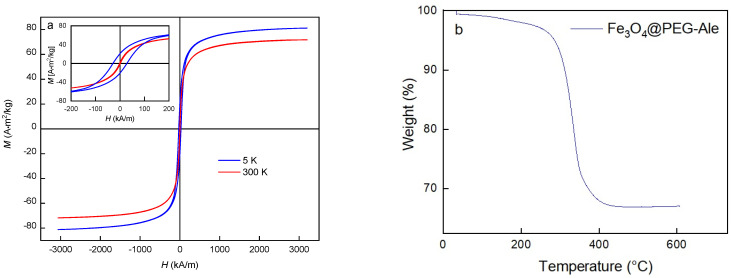

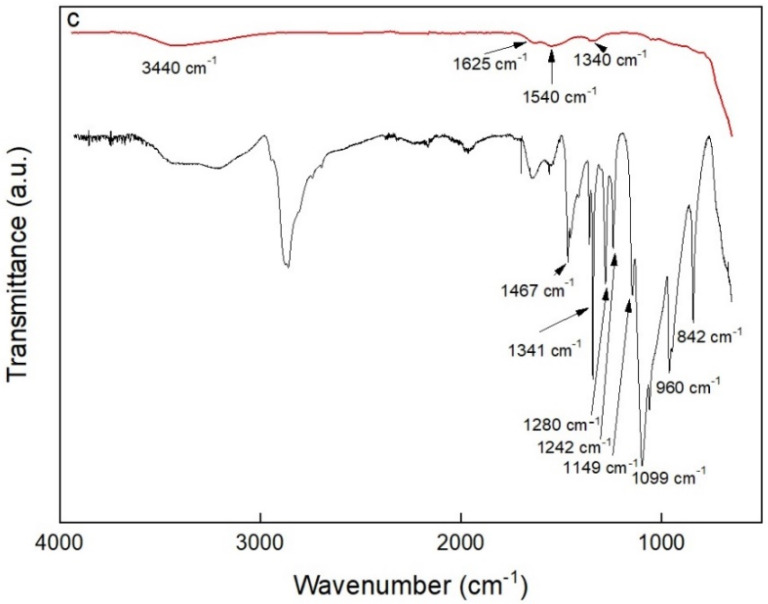
(**a**) Hysteresis loops of pure Fe_3_O_4_ nanoparticles at 5 and 300 K, (**b**) thermogravimetric analysis of Fe_3_O_4_@PEG-Ale, and (**c**) FTIR spectra of Fe_3_O_4_ (**red**) and Fe_3_O_4_@PEG-Ale nanoparticles (**black**).

Fe_3_O_4_@PEG-Ale nanoparticles (Figure 1b) exhibited the same *D*_n_ and size distribution (Figure 3b) as original Fe_3_O_4_ particles and slightly increased *D*_h_ 110 nm. However, Fe_3_O_4_ and Fe_3_O_4_@PEG-Ale particles differed in the ζ-potential, amounting to −46 and −28 mV, respectively. Superparamagnetic properties of the pure Fe_3_O_4_ nanoparticles were confirmed by measuring of hysteresis loops at 300 and 5 K (Figure 4a). Saturation magnetization at these temperatures was 71 and 81 Am^2^/kg, respectively, which is close to that of the bulk state [30], and coercivity of the Fe_3_O_4_ was 30 kA/m at 5 K and it was negligible at room temperature.

Thermogravimetric analysis of Fe_3_O_4_@PEG-Ale nanoparticles confirmed the presence of polymer coatings on the iron oxide surface, reaching 33 wt.% (Figure 4b). Moreover, the distance between the attachment points of the PEG-Ale on the particle surface (*d*) and Flory radius (*r_f_*), respectively, were calculated [31,32] from the grafting density of the polymer (0.54 chains/nm^2^) and *M*_n_ of PEG. The *r_f_*/*d* ratio characterized the PEG conformation on particle surface that can be brush-like (*r_f_*/*d* > 1) or mushroom-like (*r_f_*/*d* < 1). As the *r_f_*/*d* ratio of Fe_3_O_4_@PEG-Ale particles was 4.45, brush-like conformation of PEG was confirmed. The presence of polymer on surface of nanoparticles was also supported by FTIR (Figure 4c). The spectrum of pure Fe_3_O_4_ showed broad band at 3440 and 1625 cm^−1^ belonging to O–H stretching vibration and O–H deformed vibration, respectively, proving the presence of coordinated OH groups or water on the particle surface [33]. Peaks at 1540 and 1340 cm^−1^ were probably associated with the presence of ammonium carbonate [34] due to the reaction of air CO_2_ with ammonia during precipitation of magnetite. The spectrum of PEG-coated nanoparticles showed C–O and C–C stretching and CH_2_ rocking at 840 cm^−1^, CH_2_ rocking and twisting at 960 cm^−1^, C–O and C–C stretching at 1097 cm^−1^, C–O stretching and CH_2_ rocking at 1140 cm^−1^, CH_2_ twisting at 1241 and 1278 cm^−1^, CH_2_ wagging at 1341 cm^−1^, and CH_2_ scissoring at 1466 cm^−1^.

### 3.2. Blood Pressure, Heart Rate and Red Blood Cell Parameters

**Figure 5:**
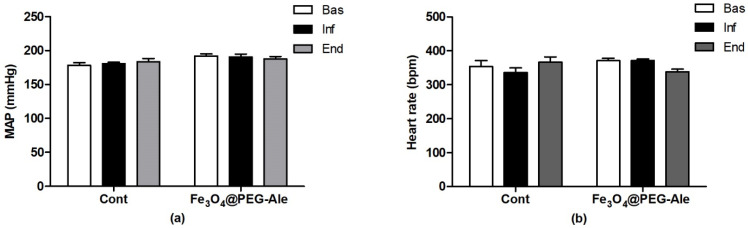
(**a**) Mean arterial pressure and (**b**) heart rate of rats 10–14 min before infusion, during infusion, and 100 min after infusion of nanoparticles. The values represent the mean ± SEM, *n* = 6–7 per group. Fe_3_O_4_@PEG-Ale, Fe_3_O_4_@PEG-alendronate nanoparticles; MAP, mean arterial pressure; HR, heart rate; Bas, basal value; Inf, value determined during infusion; End, value at the end of the experiment; bpm, beats per minute.

**Figure 6:**
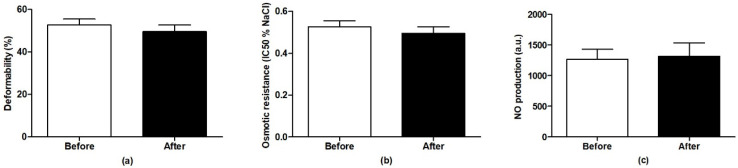
(**a**) Red blood cell deformability, (**b**) osmotic resistance, and (**c**) nitric oxide production in spontaneously hypertensive rats before and 100 min after Fe_3_O_4_@PEG-Ale nanoparticle infusion. The values represent the mean ± SEM, *n* = 3 (deformability and osmotic resistance), *n* = 5 (NO production).

MAP and HR of all rats at the beginning of the experiment were 184 ± 3 mmHg and 361 ± 10 bpm (*n* = 6–7 per group) and no significant differences between the groups were observed. Administration of Fe_3_O_4_@PEG-Ale did not alter BP and HR, neither during the infusion nor at the end of the experiment compared to the corresponding time-point in the control group (Figure 5a,b). There were no differences in RBC deformability (*n* = 3), osmotic resistance (*n* = 3) and NO production (*n* = 5) by RBCs in rats determined 100 min after administration of Fe_3_O_4_@PEG-Ale nanoparticles, compared to basal levels of these rats (Figure 6).

### 3.3. Determination of Nitric Oxide Synthase Activity

**Figure 7:**
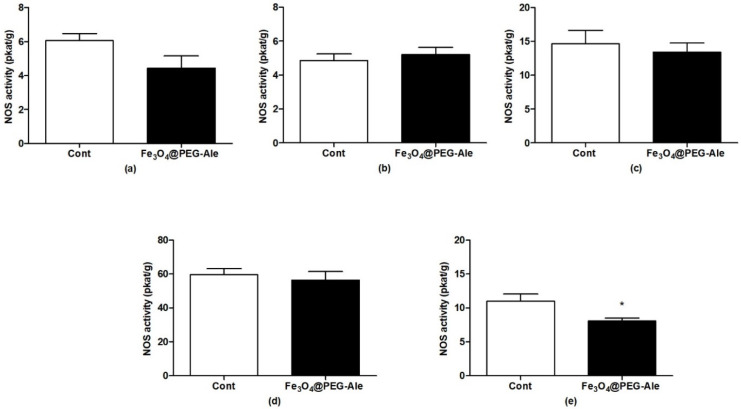
Nitric oxide synthase activities in (**a**) aorta, (**b**) left heart ventricle, (**c**) liver, (**d**) hypothalamus, and (**e**) kidney after IV administration of Fe_3_O_4_@PEG-Ale nanoparticles in rats. The values represent the mean ± SEM. * *p* < 0.05 vs. control group, *n* = 6–7 per group. Cont, control; NOS, nitric oxide synthase; Fe_3_O_4_@PEG-Ale, Fe_3_O_4_@PEG-alendronate nanoparticles.

To determine the effect of Fe_3_O_4_@PEG-Ale nanoparticles on NO production, activity of NOS was determined in the rat aorta, left heart ventricle, liver, hypothalamus and kidney (*n* = 6–7 per group). Nanoparticles had no significant effect on NOS activity in the aorta, left heart ventricle, liver, and hypothalamus (Figure 7a–d). Significant reduction of NOS activity was observed in the kidneys by ~26%, (*p* < 0.05) vs. the control group (Figure 7e). 

### 3.4. Production of Superoxide

**Figure 8:**
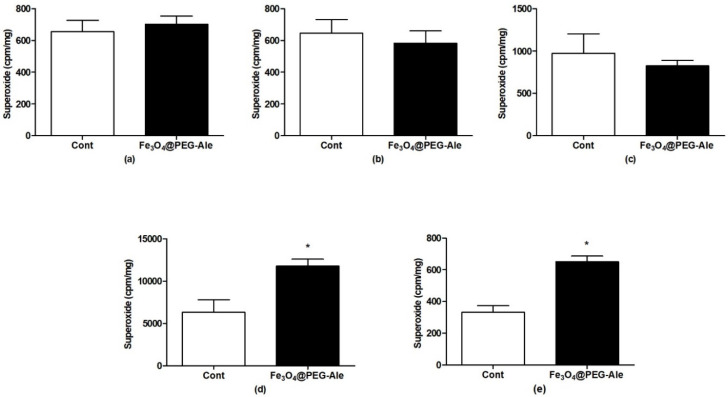
Superoxide production in (**a**) aorta, (**b**) left heart ventricle, (**c**) liver, (**d**) spleen, and (**e**) kidney after IV administration of Fe_3_O_4_@PEG-Ale nanoparticles in rats. The values represent the mean ± SEM * *p* < 0.05 vs. control group, *n* = 5–6 per group. Cont, control; Fe_3_O_4_@PEG-Ale, Fe_3_O_4_@PEG-alendronate nanoparticles.

NPs did not elevate superoxide production in the aorta, left heart ventricle and liver, (Figure 8a–c). The highest levels of superoxide in control conditions were found in the spleen, in which NPs elevated the superoxide level approximately by 86% (*p* < 0.05) vs. control (Figure 8d). Superoxide production was also significantly elevated in the kidney by ~96% (*p* < 0.05) vs. the control group (Figure 8e).

### 3.5. Examination of Contractions of the Femoral Artery

**Figure 9:**
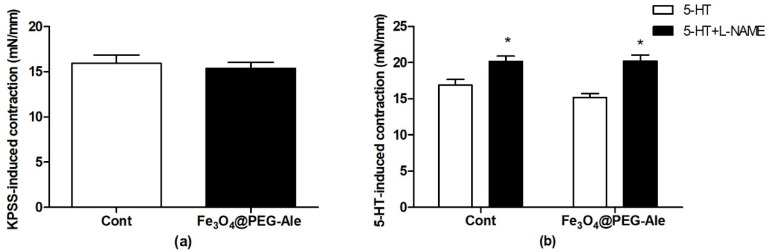
Maximal depolarization-induced contractions produced by (**a**) KPSS and (**b**) serotonin (5-hydroxytryptamine)-induced contractions in the absence and presence of NO synthase inhibitor L-NAME. The values represent mean ± SEM. * *p* < 0.05 vs. 5-HT, *n* = 12 per group. Cont, control; Fe_3_O_4_@PEG-Ale, Fe_3_O_4_@PEG-alendronate nanoparticles; KPSS, high concentration of potassium (125 mmol/L)-containing physiological saline solution; 5-HT, serotonin; L-NAME, *N*(ω)-nitro-L-arginine methyl ester.

The mean internal diameters of all arterial segments of control and Fe_3_O_4_@PEG-Ale-treated rats were 682 ± 4 and 680 ± 8 µm, respectively, and they did not differ significantly. The maximal depolarization-induced contractions produced by high concentration of potassium (125 mmol/L K^+^) in the control and Fe_3_O_4_@PEG-Ale groups did not differ significantly (Figure 9a). 5-HT-induced contractions in the absence of L-NAME were similar in the control and Fe_3_O_4_@PEG-Ale groups (Figure 9b). Pre-incubation of the arteries with L-NAME significantly enhanced the 5-HT-induced contraction of the femoral arteries in both groups investigated (*p* < 0.05 for both groups) and Fe_3_O_4_@PEG-Ale did not alter this parameter (Figure 9b).

### 3.6. Examination of Relaxations of the Femoral Artery

**Figure 10:**
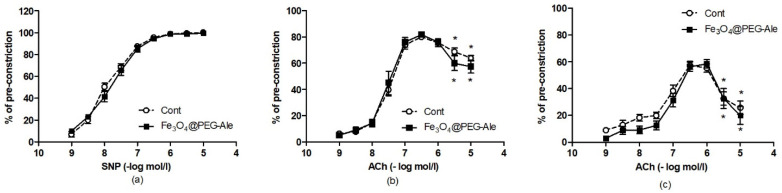
(**a**) Sodium nitroprusside-induced relaxation and acetylcholine-induced relaxations (**b**) in the absence and (**c**) presence of L-NAME in the femoral arteries. The values represent the mean ± SEM, *n* = 12 per group. * *p* < 0.05 vs. the maximal relaxation in the same group. Cont, control; Fe_3_O_4_@PEG-Ale, Fe_3_O_4_@PEG-alendronate nanoparticles, ACh, acetylcholine; SNP, sodium nitroprusside.

Sodium nitroprusside-induced and acetylcholine-induced concentration-response curves of 5-HT-precontracted femoral arteries are shown in Figure 10. SNP-induced relaxations were similar in the control and Fe_3_O_4_@PEG-Ale groups (Figure 10a). ACh-induced relaxations in the absence (Figure 10b) and presence (Figure 10c) of L-NAME were not altered by Fe_3_O_4_@PEG-Ale. Pre-treatment of the arteries with L-NAME led to significant reduction of relaxations at the highest Ach concentration in both groups investigated vs. the maximal relaxation in the given curve, suggesting no differences in the release of endothelium-derived contracting factors between control and NP-treated rats.

## 4. Discussion

In this study, superparamagnetic PEG-Ale-covered magnetite nanoparticles were synthetized. Magnetite was preferred to maghemite not only due to a one-step synthesis, but also because Fe(III) is metabolized to Fe(II) in the living organism. We also hypothesized that difference in cytotoxicity between both iron oxides is negligible. The prepared magnetite nanoparticles were coated with PEG-based polymer, that is considered to be biocompatible and bioinert and able to temporarily mask the nanoparticles against immune system and prolong their circulation in the blood stream [35]. The polymer was designed to be terminated by functional end-groups to allow a solid attachment to the iron oxide surface. In this respect, Ale appeared to be especially convenient, as its bisphosphonate groups readily conjugate to the iron oxide surface forming stable complexes [35]. Moreover, both iron oxides and Ale, as well as PEG, are FDA-approved for using in human medicine [21,22,36]. This is the advantage of Ale compared to previously used neridronate that is still waiting for final approval. Besides, Ale is easily commercially available and reasonably priced.

The increase in *D*_n_ of neat Fe_3_O_4_ and Fe_3_O_4_@PEG-Ale nanoparticles was not detected by TEM, and D_h_ was only slightly raised, probably due to the presence of poly(ethylene glycol) shell. Difference between the hydrodynamic diameter *D*_h_ and *D*_n_, was observed, with *D*_h_ being naturally higher than the number-average diameter *D*_n_ due to several reasons. First, hydrodynamic diameter can be approximated as *D*_h_ = ∑*D*_i_^6^/∑*D*_i_^5^, which provides larger numbers than *D*_n_ = ∑*D*_i_/*N* [37]. Second, DLS method is very sensitive to the presence of large particles, which also exponentially increases intensity of scattered light [38]. As a result, even a small fraction of large particles can dramatically increase the hydrodynamic diameter. The third reason is that DLS measures objects in solution, where they can aggregate and scatter light more intensively than the individual particles, while TEM determines individual particles.

Absolute value of ζ-potential decreased after PEG coating, which suggests that electroneutral PEG coating was bound to the iron oxide surface. The presence of polymer was also quantified by thermogravimetric analysis and confirmed by FTIR spectroscopy. The *r_f_*/*d* ratio > 1 confirmed that PEG-Ale was densely packed on the particle surface in a brush-like conformation. Magnetic measurement exhibited typical behavior for superparamagnetic materials; that means the nanoparticles are magnetic in an external magnetic field, but in its absence, they do not exhibit magnetism and do not aggregate as, e.g., ferromagnetic nanoparticles.

We also investigated biological effect of these NPs in hypertensive rats. The main findings are that Fe_3_O_4_@PEG-Ale (*i*) did not alter BP and HR, (*ii*) had no negative effects on fundamental RBC properties, and (*iii*) did not affect vascular function after acute intravenous administration. In addition, Fe_3_O_4_@PEG-Ale did not induce increase of superoxide and reduction in NO production in the tissue of the aorta, left heart ventricle, and liver. 

As already mentioned in the introduction, the use of various types of NP in biomedical and medical applications depends on their biocompatibility, as well as stability. In human studies, vasodilatation associated with hypotension has been observed, when certain iron oxide NPs were administered as contrast agents to improve magnetic resonance imaging [21]. Similarly, a transient decrease of BP was observed after application of poly(acrylic acid)-coated γ-Fe_2_O_3_ nanoparticles in mice [39]. No effect of PEG-coated iron oxide NPs on BP and HR was found on normotensive rats using the same experimental protocol, which is in agreement with our current finding using different NPs in SHR [8]. However, PEG-coated iron oxide NPs altered vascular function of normotensive Wistar–Kyoto (WKY) rats in terms of elevation of endothelium-dependent NO-mediated components of vasorelaxation, and partially reduced 5-HT-induced contraction. In addition, PEG-coated NPs reduced the sensitivity of VSMCs to NO in WKY rats [8]. Similar vascular changes were not present in SHR rats after application of Fe_3_O_4_@PEG-Ale in this study. Researchers also showed that iron oxide NP accumulation in endothelial cells can modify vascular function, NO bioavailability, and/or induce oxidative stress [40,41,42]. In this study, NO synthase activity and superoxide production were not changed significantly in the aorta of SHR. These findings, together with no changes in vascular functions, suggested that Fe_3_O_4_@PEG-Ale do not affect negatively the endothelium and vascular smooth muscle cells of the femoral artery in rats with high BP. Similarly, Fe_3_O_4_@PEG-Ale had no negative effects in the human umbilical vein endothelial cell cultures [43].

Another important finding suggesting good biocompatibility of Fe_3_O_4_@PEG-Ale is the fact that these NPs did not modify RBC deformability, which represents the crucial characteristic allowing RBC passage through the narrow capillaries in the microcirculation and is also a determinant of whole blood viscosity. RBC deformability is maintained by various regulatory mechanisms, among which NO production by RBCs plays an important role. In this study, NO production by RBCs was not affected by infusion of Fe_3_O_4_@PEG-Ale. In addition, Fe_3_O_4_@PEG-Ale did not modify the RBC properties to challenge the changes in osmotic pressure. Thus, the Fe_3_O_4_@PEG-Ale NPs seem to be RBC-biocompatible during in vivo conditions in SHR. In addition to the aorta, Fe_3_O_4_@PEG-Ale did not induce elevation in superoxide production in the tissues of the left heart ventricle and liver. On the other hand, Fe_3_O_4_@PEG-Ale elevated superoxide production in the kidney and spleen. This may be related to the fact that NPs are excreted by the kidneys and/or internalized in the spleen. Our findings are in contrast to elevated superoxide production in the liver, aorta and left heart ventricle found in normotensive rats using PEG-coated NPs [44]. We assume that the differences can result mainly from different hemodynamic situation in SHR, as well as from size and different physicochemical properties of NPs. 

NO is the main vasorelaxant molecule in the cardiovascular system, but serves as a neurotransmitter and neuromodulator in organisms. In this study, reduced NO production was found only in the kidneys, together with elevated superoxide production. This finding is similar to findings in the kidney of WKY rats using PEG-coated NPs without Ale [8,44]. On the other hand, our findings suggested that Fe_3_O_4_@PEG-Ale NPs produced less changes in the cardiovascular tissue, liver and hypothalamus than previously used PEGylated NPs, which may result from different physicochemical properties, size and/or modified coating. In hypertensive rats, an important role may be played by altered hemodynamic state (blood pressure and blood flow), which may accelerate NPs clearance from circulation. However, independently of differences in above mentioned factors (hemodynamic situation, physicochemical properties, and coating of NPs), reduction of NO and elevated superoxide in the kidneys, might suggest at least partial NOS uncoupling resulting in oxidative stress. As oxidative damage might later be followed by functional and/or structural changes in the tissues, attention should be paid to the possible harmful effect of NPs to kidneys. 

In conclusion, we prepared superparamagnetic magnetite NPs with *D*_n_ = 11 nm covered with PEG-Ale coating, and moderately narrow size distribution, for possible use as an agent increasing MRI contrast. Determination of biological influences of Fe_3_O_4_@PEG-Ale NPs did not show negative effects on the cardiovascular system and fundamental RBC parameters after acute intravenous administration in SHR. Fe_3_O_4_@PEG-Ale NPs induced increase in superoxide and reduction in NO production in the kidney. Thus, despite that there were no significant effects of Fe_3_O_4_@PEG-Ale on the cardiovascular system and RBCs, further studies are needed to evaluate their effect in the kidneys. These findings contribute to complex knowledge about behavior of magnetic nanoparticles in in vivo animal models, considering also the influence of high BP, which makes this paper valuable in terms of nanotoxicology research.

## Data Availability

The data presented in this study are contained within the article.

## Abbreviations

5-HT	5-hydroxytryptamine (serotonin)
ACh	acetylcholine
Ale	alendronate
ANOVA	analysis of variance
Bas	baseline
BP	arterial blood pressure
bpm	beats per minute
Cont	control
*Ð*	dispersity
*D*_h_	hydrodynamic diameter
DLS	dynamic light scattering
*D*_n_	number-average diameter
*D*_w_	weight-average diameter
FDA	Food and Drug Administration
Fe_3_O_4_@PEG-Ale	magnetite coated with poly(ethylene glycol)-alendronate
FTIR	Fourier-transform infrared
HR	heart rate
i.v.	intravenous
KPSS	high concentration of potassium (125 mmol/l)-containing physiological saline solution
L-NAME	*N*(ω)-nitro-L-arginine methyl ester
MAP	mean arterial pressure
*M*_n_	number-average molar mass
*M*_w_	weight-average molar mass
MRI	magnetic resonance imaging
NMR	nuclear magnetic resonance
NO	nitric oxide
NOS	nitric oxide synthase
NPs	nanoparticles
PB	phosphate buffer
*PD*	polydispersity
PEG	poly(ethylene glycol)
PEG-NHS	α-methoxy poly(ethylene glycol) succinimidyl ester
PSS	physiological saline solution
RBC	red blood cell
SEM	standard error of mean
SHR	spontaneously hypertensive rat
SNP	sodium nitroprusside
TEM	transmission electron microscope
TGA	thermogravimetric analysis
WKY	Wistar-Kyoto rat
ZFC-FC	zero-field-cooled and field-cooled
